# Family Networks, Social Networks, and Life Satisfaction of Older Adults in China

**DOI:** 10.3390/healthcare10081568

**Published:** 2022-08-18

**Authors:** Weisong Cheng, Wenhao Song, Chunhui Ye, Zhonghan Wang

**Affiliations:** 1 China Academy for Rural Development, Zhejiang University, Hangzhou 310058, China; 2 School of Public Affairs, Zhejiang University, Hangzhou 310058, China

**Keywords:** family networks, social networks, physical mistreatment, emotional mistreatment, life satisfaction of older adults

## Abstract

Older adults’ family networks and social networks are important factors that influence life satisfaction, but their transmission mechanisms have not been adequately discussed. The objective of this study was to examine the mechanisms through which family networks and social networks influence the life satisfaction of older adults. We empirically examined the effects and mechanisms of older adults’ family networks and social networks on their life satisfaction using the 2018 China Longitudinal Aging Social Survey with a sample size of 11,418 older adults aged 60 years and older. In the research sample, 6.47% of older adults were subjected to at least one form of mistreatment. The research results indicate that family networks (β = 0.0060, *p* < 0.05) and social networks (β = 0.0122, *p* < 0.01) have a significant positive effect on older adults’ life satisfaction. The mechanism-of-action test found that family networks and social networks enhance older adults’ life satisfaction, mainly by reducing the level of physical mistreatment they experience, but these networks cannot improve the life satisfaction of the elderly by reducing their emotional mistreatment. Further research found that community-provided medical escorts, home chores, and meal delivery services can all alleviate the decline in life satisfaction among older adults due to emotional mistreatment. This study deepens our understanding of how older adults’ family and social networks affect their life satisfaction as we examine the mediating role of the physical and emotional mistreatment of older adults and discuss the effects of potential policy interventions.

## 1. Introduction

Aging has become an important issue affecting the economic and social development of countries around the world. According to the 2019 Revision of World Population Prospects, by the middle of the 21st century, the proportion of the world’s elderly population will exceed 16% [1]. By that time, one in six people worldwide will be 65 years old or older. As older adults represent an important segment of the population, their quality of life is important because it affects the well-being of all of society [2]. In developing countries such as China, the aging rate is faster and more serious. According to the World Bank, China was forecasted to have the largest share of people aged 65 and over in the world by 2020, at 23.34%. However, society as a whole provides limited welfare for the elderly due to the level of economic development and the social security system [3]. Therefore, the overall quality of life of older people in China is not high. More worryingly, the relative quality of life of older people in other developing countries is lower than in China [4]. The World Health Organization defines quality of life as “an individual’s perception of their position in life in the context of the culture and value systems in which they live and in relation to their goals, expectations, standards and concerns” [5]. According to this definition, life satisfaction indicators have received widespread attention from scholars as a basic measure of the quality of life of older adults because they take into account people’s different responses, interpretations, and adjustments to external social conditions [6,7]. Therefore, it is important and meaningful to focus on life satisfaction of older adults in China.

In order to improve the life satisfaction of older adults, we need to identify influential factors. Academics generally agree that income level, health level, accessibility to health services, and family and community environment affect older adults’ life satisfaction [8,9,10,11]. Among these factors, health factors have received increasing attention from scholars [12], especially mental health factors such as emotional state and depression, which tend to have a significant impact on the life satisfaction of older adults. Studies have shown that the mental health of some older adults is in a long-term suboptimal healthy or unhealthy state, and this unhealthy state is closely related to isolation, neglect, and mistreatment from the external environment [13,14]. In China, the suicide rate among people over 65 years of age is four to five times higher than that among the general population, reaching 44.3–200 per 100,000 people, demonstrating the negative outcomes of poor long-term mental health [15,16]. In addition, various forms of violence and mistreatment against older adults are prevalent. According to the United Nations Population Fund, 9.4% of rural older women and 5% of urban older women have experienced physical and mental mistreatment. Studies have found that older adults who have been physically and mentally mistreated show a precipitous, long-term decline in life satisfaction [17,18].

In addition, the relationship between the family and community environment and life satisfaction in older adults has received widespread academic attention [9,19]. Studies have found that an increase in the breadth and depth of family and social networks can enhance life satisfaction among older adults [20,21,22]. However, research on the mechanisms of influence between the two is somewhat lacking. Recent literature has found that the development of family and social networks can alleviate the physical and mental mistreatment of older adults to some extent [23]. That said, can family networks and social networks enhance the life satisfaction of older adults by reducing their physical and psychological mistreatment? We conducted an empirical study on this question using the 2018 CLASS database of the Renmin University of China in order to provide feasible ideas for improving the life satisfaction of older adults.

The marginal contributions of this study are as follows. First, it expands the channels of influence of family networks and social networks on the life satisfaction of the elderly through the test of mediating mechanisms. Second, it focuses on elder mistreatment, tests it empirically, and distinguishes two different forms of mistreatment, i.e., physical mistreatment and emotional mistreatment. Third, an exploratory attempt is made to study the potential role of existing community services in mitigating the negative effects of the emotional mistreatment of the elderly.

## 2. Literature Review and Research Hypothesis

### 2.1. Family Network, Social Network, and Life Satisfaction

Life satisfaction is an important positive psychological outcome. Life satisfaction is an overall judgment of one’s life experiences and is an important component of subjective well-being. Lawton and Nahemow first proposed the Ecological Theory of Aging (ETA) in 1973, which was developed and refined by Lawton (1977), Rowles (1983), and others, and gradually formed a complete theoretical framework [24,25,26]. In the Ecological Theory of Aging (ETA), aging is a critical stage in one’s life course that is profoundly influenced by the physical environment; this has the potential to both constrain and increase opportunities for aging, where an individual’s optimal level of functioning depends on a unique combination of personal capabilities and environmental characteristics [27] and where the subjective well-being of older adults is a function of their personal and environmental characteristics [28]. Thus, family networks and social networks, as environmental characteristics, can be conceptualized as sources of positive effects on the well-being of older adults. In particular, recent literature suggests that the breadth and depth of family networks and social networks have a positive impact on life satisfaction by mitigating elder mistreatment [29,30].

However, these studies do not distinguish between the different effects of the breadth and depth of family networks and social networks on life satisfaction. Specifically, the family network refers to emotional involvement among family members and the social network refers to emotional involvement in social interactions. The breadth of both refers to how many nodes of long-term connections are present, while depth refers to the extent to which emotional involvement is constructed. A recent study suggests that although both family networks and social networks have important effects on life satisfaction, their effects on psychological outcomes and physical health may differ and should be studied separately [31,32]. For older adults, several studies in Western societies have shown that only family networks are significantly associated with physical and emotional mistreatment, while social networks are not [33,34]. However, this finding may be biased due to the limitations of previous study designs. For example, several studies have used data from the National Social Life Health and Aging Project (NSHAP), which suffers from over-sampling and missing sample interpolation. In addition, due to the popularity of Confucian culture regarding family filial piety, there may be heterogeneity in the effects of family networks and social networks on life satisfaction. In other words, the results of studies in Western countries may not be fully consistent with those in Asian countries. However, in the context of Chinese society, few studies [9] have examined the different effects of family networks and social networks on life satisfaction.

In addition, according to the Ecological Theory of Aging, older adults are in a family environment and a social environment and are influenced by their interpersonal networks [35]. Therefore, elder mistreatment and neglect may be mitigated by the extensiveness of family networks and social networks. Discussing potential mechanisms to mitigate elder physical and emotional mistreatment may have positive implications for prevention and intervention. Therefore, we investigated such potential pathways of influence to inform future policy development.

### 2.2. Physical Mistreatment and Life Satisfaction in Older Adults

Elder physical mistreatment is the unreasonable physical treatment of an older person, i.e., family members use their body strength to subject the older person to illness, pain, injury, or death. For older adults, physical mistreatment has a direct impact on their life satisfaction. According to the Ecological Theory of Aging, individuals determine their life satisfaction based on the degree of injustice they have experienced in their lives. Therefore, the physical mistreatment of older adults has a significant impact on their life satisfaction. In general, older adults who have suffered physical mistreatment may have serious negative evaluations of life that are detrimental to their personal well-being. Older adults who do not experience physical mistreatment have a higher overall evaluation of life. Specifically, physical mistreatment can cause serious physical and psychological harm to older adults: physically, physical mistreatment puts older adults at risk of disability and premature death; psychologically, physical mistreatment can have long-term negative psychological consequences, such as depression and anxiety [36]. Dual physical and psychological harm has a direct negative impact on the life satisfaction of the elderly.

### 2.3. Emotional Mistreatment and Life Satisfaction in Older Adults

Elder emotional mistreatment can be regarded as mistreatment caused by the deliberate neglect of the emotional needs of the elderly by their main caregivers. In the Ecological Theory of Aging, the neglect of older adults by primary caregivers is considered as mistreatment of older adults in the home environment or social environment, which can adversely affect the mental health of older adults and, thus, reduce their life satisfaction. In general, older adults who experience emotional mistreatment may have a severely negative perception of life that is detrimental to their personal well-being. Older adults who do not experience emotional mistreatment have a higher overall evaluation of life. Specifically, older adults who experience emotional mistreatment may perceive themselves as unappreciated and have their communication needs ignored by their primary caregivers, which leads to negative perceptions of social interactions and increases social distance from others. Thus, elder emotional mistreatment may lead to decreased life satisfaction in older adults. Numerous studies have shown that elder emotional mistreatment predicts negative psychological outcomes, such as anxiety, depression, and phobias [37,38]. In addition, one study found that Chinese American older adults who were mistreated were more likely to experience anxiety symptoms and depressive symptoms, which were thought to be closely related to violations of traditional Chinese social values [38]. Chronic negative psychological states have a significant negative effect on life satisfaction in older adults.

### 2.4. Other Factors Affecting Life Satisfaction of Older Adults

In addition to factors that have an impact on the life satisfaction of older adults such as family networks, social networks, and physical and emotional mistreatment, several other factors could potentially play a role. Gender and age as basic characteristics have received extensive attention in many studies [39,40], and there are differences in life satisfaction among older adults of different ages and genders, e.g., Macia et al. [39] found that with advancing age, older adults presented higher life satisfaction; older women had higher life satisfaction than older men. In addition, being married or widowed has an important effect on older adults’ life satisfaction [41,42], and older adults who are widowed have lower life satisfaction [43]. Some studies have found that race and religion have heterogeneous effects on older adults’ life satisfaction [44,45], older adults of Chinese descent have lower life satisfaction [46], and older adults with a higher sense of religious meaning have higher life satisfaction [45]. In China, the “peasant” status to which one belongs if they have a rural hukou confers lower resource endowment, which reduces the life satisfaction of rural older adults [11]. In addition, older adults with higher levels of education and those with higher levels of health have higher life satisfaction [47,48,49]. The longer they have lived in the county, the stronger the ties of family and friends of the elderly and the higher their identification with the community, raising their life satisfaction [9].

### 2.5. Family Networks, Social Networks, and Physical and Emotional Mistreatment in Older Adults

It is worth noting that the development of family networks and social networks has a potential mitigating effect on elder mistreatment. In terms of emotional mistreatment, the extension of family networks and social networks can help alleviate the emotional distress of older adults and help them increase the interpersonal support, which may effectively reduce the probability of experiencing unfair emotional treatment or self-neglect [50]. Both cross-sectional and longitudinal studies have confirmed this potential impact. For example, a study by Burnett et al. [51] showed that the development of family networks and social networks helped to alleviate self-neglect in older adults and slowed the onset of depression. In terms of physical mistreatment, the development of family and social networks can provide additional social support for older adults, and the additional support or intervention reduces the likelihood of physical mistreatment by the older adult’s primary caregiver because the older adult has more resources to seek outside help. Conversely, older adults are more likely to experience physical mistreatment if their family and social networks tend to shrink [52]. The above review shows potential mechanisms to hypothesize that elder mistreatment may be mitigated by the support of family networks and social networks, which, in turn, enhances their life satisfaction.

**Figure 1:**
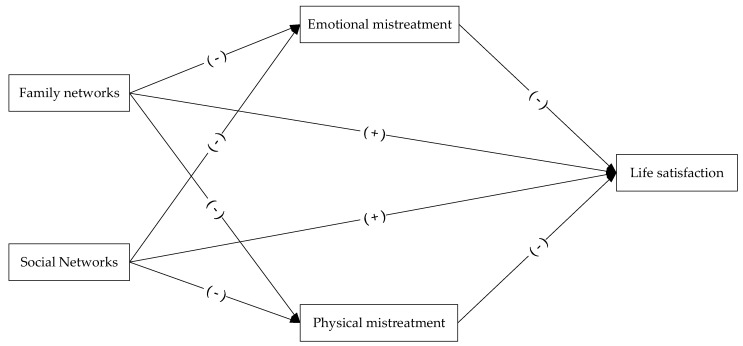
Conceptual framework.

Previous studies have shown that the physical and emotional mistreatment of elders have a significant negative impact on life satisfaction. However, few studies have focused on the potential ways to alleviate elder physical and emotional mistreatment. In China, under the influence of Confucian culture, family relationships are regarded as an important part of intimate relationships, and the extension of family networks may have a potential role in alleviating elder physical and emotional mistreatment. In addition to this, older adults living in the same area for a long time have fixed social networks, and social networks may have significant intervention effects on elder physical and emotional mistreatment. Therefore, in this paper, we discuss the effects of family and social networks on the life satisfaction of older adults in mitigating their mistreatment in the Chinese social context through a large, nationally representative sample. As a result, the following three sets of hypotheses are proposed. In addition, we have drawn a conceptual framework based on the hypotheses (see Figure 1).

**Hypothesis** **1a** **(H1a):** *Family networks have a positive effect on life satisfaction.*

**Hypothesis** **1b** **(H1b):** *Social networks have a positive effect on life satisfaction.*

**Hypothesis** **2a** **(H2a):** *Elder emotional mistreatment has a negative impact on life satisfaction, and family networks can improve their life satisfaction by mitigating emotional mistreatment.*

**Hypothesis** **2b** **(H2b):** *Elder physical mistreatment has a negative impact on life satisfaction, and family networks can improve their life satisfaction by mitigating physical mistreatment.*

**Hypothesis** **3a** **(H3a):** *Elder emotional mistreatment has a negative impact on life satisfaction, and social networks can improve their life satisfaction by mitigating emotional mistreatment.*

**Hypothesis** **3b** **(H3b):** *Elder physical mistreatment has a negative impact on life satisfaction, and social networks can improve their life satisfaction by mitigating physical mistreatment.*

## 3. Materials and Methods

### 3.1. Data and Sample

A sample of 11,418 older adults was obtained for analysis using the 2018 China Longitudinal Aging Social Survey (CLASS) database from the Renmin University of China. The original study used a multi-stage probability sampling method. The age range of these older adults was 60–108 years, with an average age of 71.45 years. Descriptive statistics are given in Table 1.

### 3.2. Variable Definition

#### 3.2.1. Dependent Variable

Life satisfaction as a dependent variable was measured using a question from CLASS 2018, i.e., “Overall, are you satisfied with your current life?” This single measure was found to be reliable in previous studies on China’s elderly population [53]. Using a five-point Likert scale from 1 = very dissatisfied to 5 = very satisfied, a high score indicated a high level of life satisfaction.

#### 3.2.2. Independent Variables

The independent variables in this paper were family network and social network, which were measured mainly using a streamlined version of the Social Network Scale developed by Lubben et al. [54] (Lubben Social Network Scale 6, LSNS-6), which is used to measure the family networks and social networks of older adults, screen for people at high risk of social isolation, and assess social integration. LSNS-6 is a bidimensional scale that includes 3 items referring to family networking and 3 items referring to social networking (see Table 2 for specific questions and assignments), and the scores of the questions under each indicator are summed to the total score of the indicator, with higher scores indicating better social or family networks.

The scale has been shown to have good reliability and good validity, and is widely used as a standard scale for measuring family networks and social networks. This study analyzed the reliability of the scale and showed that it has good internal consistency (Cronbach’s alpha coefficients of 0.815 and 0.840 for the family network subscale and social network subscale, respectively).

#### 3.2.3. Mediators

Emotional mistreatment and physical mistreatment are two distinct components of elder mistreatment. Therefore, it is necessary to measure emotional and physical mistreatment separately. In this study, physical mistreatment refers to experiencing excessive control or physical violence, while emotional mistreatment refers to distress caused by verbal or nonverbal behaviors. In the CLASS 2018 questionnaire, measures of physical mistreatment include (E) “Your family member does not provide you with a regular place to live”, (F) “Your family member doesn’t feed you well/makes you eats poorly”, and (G) “Your family member does not allow you to leave the house”. Measures of emotional mistreatment include (A) “Your family member does not visit, greet/talk to you for a long time”, (C) “Your family member does not take care of you when needed”, and (D) “Your family member insults/mistreats/threatens/assaults you”.

Respondents were asked if their family member had committed any of the above mistreatments against them in the past year. Each item was coded as either 0 = did not experience or 1 = experienced. The scores for physical and emotional mistreatment were summed separately, with higher scores indicating higher levels of mistreatment.

#### 3.2.4. Control Variables

A number of variables were controlled for in this study, including age; gender (1 = male, 0 = female); marital status (1 = married, 0 = single, divorced, or widowed); religion (1 = religious, 0 = no religion); ethnicity (1 = Han, 0 = minority); hukou (1 = agricultural, 0 = non-agricultural), which is a special identification in China (where people are distinguished as non-agricultural hukou or agricultural hukou. Agricultural hukou holders experience lower status and less welfare protection); education level (from 1 = no education to 5 = university or higher), and self-rated health status (from 1 = very unhealthy to 5 = very healthy).

### 3.3. Method

First, descriptive statistics and correlation analyses were performed using stata15.0 and the underlying impact mechanism (Hypothesis 1) was tested using ordinary least squares, as shown in Equation (1):

(1) $$Y_{i}=\alpha +S_{i}+\Sigma \beta X_{i}+\varepsilon_{i}$$

where $Y_{i}$ is the dependent variable, representing the life satisfaction of the elderly, and $S_{i}$ is the key independent variable, representing the degree of family network or social network. $X_{i}$ is the control variable, and $\varepsilon_{i}$ is the random error term. Equation (1) was used to test Hypothesis 1 (family networks and social networks have a positive effect on life satisfaction).

Based on the estimation of the results using ordinary least squares, the equations were then tested for robustness using ordinal logistic regression.

Then, a mediating effect model was used to test the mediating mechanism of family social network on life satisfaction by mitigating elder mistreatment, as shown in the following equations:

(2) $$Y_{i}=i_{1}+cS_{i}+\Sigma d_{1}X_{i}+\varepsilon_{i}$$

(3) $$M=i_{2}+aS_{i}+\Sigma d_{2}X_{i}+\varepsilon_{i}$$

(4) $$Y_{i}=i_{3}+c^{\prime }S_{i}+bM+\Sigma d_{3}X_{i}+\varepsilon_{i}$$

where *M* is a mediating variable and is a measure of the extent of elder mistreatment, specifically containing emotional and physical mistreatment. The other variables remain consistent with Equation (1). The total effect of family social network on life satisfaction of the elderly is *c*, the direct effect is *c′*, and the indirect effect is *ab*. The significance test for the product of coefficients *ab* was achieved via the Bootstrap method. If the effect of family social network on the life satisfaction of the elderly does have a partial effect through the mediating variable *M*, the following two conditions must be satisfied simultaneously: (i) the variable$\;S_{i}$ has a significant negative effect on the mediating variable *M*, i.e., *a* < 0; (ii) the decrease in the mediating variable *M* promotes an increase in the life satisfaction of the elderly, i.e., *b* < 0, and the regression coefficient of the family social network variable $S_{i}$ decreases after the introduction of the mediating variable, i.e., $c^{\prime }<c$. Equations (2)–(4) are used to test Hypotheses 2 and 3.

## 4. Results

### 4.1. Preliminary Analysis

Correlations for all the variables are presented in Table A1. Bivariate correlations between the key variables indicate that family network was negatively correlated with physical mistreatment (r = −0.048, *p* < 0.01), negatively correlated with emotional mistreatment (r = −0.016, *p* < 0.1), and positively correlated with life satisfaction (r = 0.017, *p* < 0.1). Social networks were significantly negatively correlated with physical mistreatment (r = −0.035, *p* < 0.01), negatively but not significantly correlated with emotional mistreatment (r = −0.009, *p* > 0.1), and positively correlated with life satisfaction (r = 0.068, *p* < 0.01). In addition, physical mistreatment and emotional mistreatment were positively correlated (r = 0.739, *p* < 0.01), which indicates that physical and emotional mistreatment often occur together. Finally, both physical and emotional mistreatment were negatively correlated with life satisfaction (r = −0.040, *p* < 0.01; r = −0.051, *p* < 0.01).

On the basis of the correlation analysis, we first conducted a test of Hypothesis 1 (social networks and family networks have a positive effect on the life satisfaction of the elderly), and the method used was the ordinary least squares method of Equation (1). The test results are shown in Table 3.

In Table 3, after controlling for age, gender, marriage, religion, ethnicity, hukou, education level, and self-rated health, family network significantly increased the life satisfaction of older adults (b = 0.0060, *p* < 0.05) and social network had similar results (b = 0.0122, *p* < 0.01), which validates Hypothesis 1. Among the covariates, marriage, religion, ethnicity, hukou, education, self-rated health level, and years of residence in the district and county all significantly affected the life satisfaction of older adults, but other variables, such as age and gender, had no significant effect on life satisfaction.

However, ordinary least squares has difficulties in interpreting discontinuous variables, and to alleviate this problem, ordinal logistic regression was used next for robustness testing, as shown in Table 4. In Table 4, the coefficients shown are the marginal treatment effect coefficients, estimated using ordinal logistic regression.

Table 4 shows that family networks have less influence on the life satisfaction of older adults compared to social networks. Taking the column with a value of 5 as an example, for the elderly with the highest level of life satisfaction, an increase of one unit in the family network relationship can only make life satisfaction increase by 0.26%, while an increase of one unit in the social network relationship can make life satisfaction increase by 0.41%, so social networks have a greater influence on life satisfaction.

As far as the other variables are concerned, the effects of age and gender on life satisfaction are not significant. One possible explanation is that life satisfaction, as a subjective evaluation of one’s own well-being, is difficult to change due to differences in age and gender. In other words, older adults who are male or female, and older or relatively younger, have different life expectations based on their age and gender, resulting in little difference in their overall life satisfaction. Interestingly, marriage, religion, ethnicity, household registration, education, health, and years of residence in the county had significant effects on life satisfaction. As shown in Table 4, being married, having religious beliefs, being Han Chinese, having a higher level of education, having a higher level of health, and having an increased length of residence in the county increase the life satisfaction of the elderly, while having an agricultural household registration decreases the life satisfaction of the elderly. Among the variables, health level is the most influential, and the length of residence in the county is the least influential.

### 4.2. Hypothesis Testing of the Intermediary Model

On the basis of univariate regression analysis, Hypotheses 2 and 3 were tested using a mediating effects model, which state that family networks and social networks significantly enhance the life satisfaction of older adults by reducing their physical and emotional mistreatment. Hypothesis 2 was tested first, and the results are shown in Table 5.

In Table 5, the channel through which family networks increase life satisfaction is mainly the reduction in physical mistreatment, while the effect of family networks on the reduction in emotional mistreatment is not significant. Specifically, the first column of Table 5 demonstrates a significant positive effect of family networks on life satisfaction, which is the total effect of family networks on enhancing life satisfaction. The second and third columns of Table 5 demonstrate the indirect effect of family networks on enhancing life satisfaction through the reduction in physical mistreatment (coefficient b = 0.0815 × 0.0042 = 0.0003 (*p* < 0.01), accounting for approximately 6% of the total effect). In addition, the third column of Table 5 demonstrates that the effect of family networks on life satisfaction is significant at the 5% level when the channel of physical mistreatment is stripped away, and the coefficient decreases compared to the first column, suggesting that one of the channels through which family networks enhance the life satisfaction of older adults is the reduction in the physical mistreatment of older adults. Furthermore, the fourth and fifth columns of Table 5 show the indirect effect of family network on enhancing life satisfaction through the reduction in emotional mistreatment (coefficient b = 0.0019 × 0.1040 = 0.0002 (*p* > 0.1), which is about 3.2% of the total effect), but this channel of effect is not significant. Taken together, the positive effect of the family network on the life satisfaction of older adults was mainly achieved by reducing the physical mistreatment of older adults, and it was difficult for the family network to have a sufficient effect on the emotional mistreatment of older adults.

In Table 6, the channel through which social networks improve life satisfaction is, again, primarily a reduction in physical mistreatment, while the effect of social networks on the reduction in emotional mistreatment is similarly insignificant. Specifically, the first column of Table 6 demonstrates a significant positive effect of social networks on life satisfaction, which is the total effect of social networks on enhancing life satisfaction. The second and third columns of Table 6 demonstrate the indirect effect of social networks on life satisfaction through the reduction in physical mistreatment (coefficient b = 0.0028 × 0.0786 = 0.0002 (*p* < 0.01), accounting for approximately 1.8% of the total effect). In addition, the third column of Table 6 demonstrates that the effect of social networks on life satisfaction is significant at the 1% level when the channel of physical mistreatment is stripped away, suggesting that one of the channels through which social networks enhance the life satisfaction of older adults is the reduction in the physical mistreatment of older adults. Furthermore, the fourth and fifth columns of Table 6 show the indirect effect of social networks on enhancing life satisfaction through the reduction in emotional mistreatment (coefficient b = 0.0003 × 0.1050 = 0.00003 (*p* > 0.1), which is about 0.3% of the total effect), but this channel of effect is not significant. Taken together, one of the channels of influence of social networks on older adults’ life satisfaction is the reduction in the physical mistreatment of older adults, and it is equally difficult for social networks to have a sufficient effect on the emotional mistreatment of older adults.

**Figure 2:**
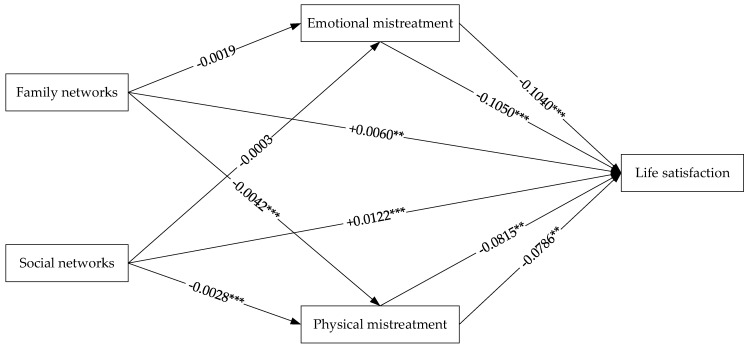
Path analysis results; the numbers are the unstandardized regression coefficients obtained from the ordinary least squares; ** 5% significance, *** 1% significance.

Based on the above analysis, the results presented in Figure 2 are those of the path analysis. In Figure 2, both family networks and social networks had no significant effect on emotional mistreatment, both had a significant effect on physical mistreatment, and both enhanced life satisfaction by mitigating physical mistreatment.

In response to the emotional mistreatment of older adults that cannot be alleviated through family networks and social networks, some community services may be able to provide relief if they are offered. As shown in Table 7, the decline in the life satisfaction of older adults due to emotional mistreatment would be mitigated if the community provided escorted medical appointments, home-based chores, and meal delivery services. On the one hand, the services provided by the community help to replace the absence of care by the direct caregiver, and on the other hand, some of the services provided by the community are long-term in nature and help to observe the living conditions and emotional performance of the elderly in a timely manner; this can effectively alleviate the emotional mistreatment of the elderly. However, it is worth noting that community-based counseling does not show similar effects. One possible explanation is that counseling services make older adults defensive, and under the influence of traditional thinking that “family shame should not be disclosed”, older adults are reluctant to share their experiences of emotional mistreatment with psychologists, so the impact of counseling is weak. In contrast, other daily care services provided by the community can effectively reduce the defensiveness of older adults and, thus, have a significant effect on the emotional mistreatment of older adults.

## 5. Discussion

Current research remains sparse on reducing the channels through which elder mistreatment deteriorates life satisfaction. Based on a large sample, with this study, we aimed to expand the existing research related to elder mistreatment in mainland China. This study is a preliminary attempt to explore channels that might reduce elder mistreatment and to enhance their life satisfaction; this was achieved by using a mediating effects model that focused on factors that influence the life satisfaction of older adults, and the findings are discussed as follows.

First, both family networks and social networks have a direct positive effect on the life satisfaction of older adults in China. This result is consistent with previous empirical studies in Western societies, indicating that the expansion of family networks and social networks leads to the improvement of older adults’ quality of life. In traditional Chinese culture, the social and family networks of older adults are effective in improving their moods and encouraging them to go out and be active, thus, effectively enhancing their life satisfaction.

Second, among the other factors affecting the life satisfaction of the elderly, age and gender have no significant effects, indicating that there are no differences in life satisfaction when older males are compared to older females, for instance. In addition, having a spouse can make the life satisfaction of the elderly increase; with the presence of an elderly partner, they can not only chat and relieve boredom, but they also can take care of each other, which greatly enhances the life satisfaction of the elderly. Third, the life satisfaction of older adults with religious beliefs is enhanced. For example, in recent years, Buddhism, which has become increasingly popular in rural China, has provided psychological comfort and practical help to believers through meetings and rituals, effectively enhancing the life satisfaction of older adults. In terms of ethnicity, because Han Chinese is the main ethnic group in China, the cultural environment and lifestyle are dominated by the Han lifestyle. Therefore, the life satisfaction of the elderly of Han ethnicity is higher. In terms of hukou, China’s agricultural hukou confers a lower social status and fewer social benefits to rural older adults, which limits their life satisfaction. In terms of education, older adults with higher levels of education have higher life satisfaction due to better adaptive interaction with their environment. In terms of health, older adults with higher levels of health have higher satisfaction due to physical comfort and happiness. The longer they live in an area, the more fully developed their social network is and the higher their sense of local identity, which contributes to the life satisfaction of the elderly. Among all the factors, the health factor has the greatest influence on life satisfaction, indicating that health is the foremost concern among the elderly and is the core of senior life.

Third, the results of the mediating effect test showed that both family networks and social networks were effective in reducing the physical mistreatment of older adults, which, in turn, was effective in improving their life satisfaction. However, neither family networks nor social networks had a significant effect on emotional mistreatment among older adults. The possible explanation for this finding is that, whether it is from a close family member or a close friend, under the influence of Confucianism, emotionally mistreated older adults do not perceive emotional mistreatment, on the one hand, and are reluctant to seek help from the outside world, on the other. In addition, from the perspective of family and social networks, emotional mistreatment is more difficult to detect than physical mistreatment; moreover, it is more hidden, and the strained emotional relationship between the elderly and their immediate caregivers is less visible to “outsiders”. Thus, the decrease in physical mistreatment was significant, but the decrease in emotional mistreatment was not.

Comparing the effects of the mediating variables of family network and social network, the effects of both family network and social network on life satisfaction were found to be achieved by reducing the physical mistreatment of older adults. However, for the effect on older adults’ life satisfaction, social networks were more influential than family networks. For the effect on physical mistreatment, social networks were less influential than family networks, which made social networks less influential than family networks in enhancing life satisfaction by reducing the physical mistreatment of older adults. In other words, family networks are more significant and effective in reducing domestic violence because of the closeness of family ties, and social networks have a limited role in this regard. Where social networks exert an effect is through other channels of enhancing older adults’ life satisfaction, such as interpersonal interactions and outings, which can have a greater and broader effects on older adults’ life satisfaction.

Fourth, this paper is inconsistent with prior literature, which found that both physical and emotional mistreatment led to decreased life satisfaction in older adults. The correlation between the occurrence of physical and emotional mistreatment was strong, and both forms of mistreatment had a significant effect on negative emotional symptoms, leading to a decrease in life satisfaction. The reason for the different results from the previous literature may be that previous studies excluded too many samples, which resulted in a severe loss in sample representativeness. In the present study, the results were re-tested using a representative sample of more than 10,000 cases, with the exclusion of a small number of samples to enhance the credibility of the test results.

Fifth, in terms of mitigation methods for the emotional mistreatment of the elderly, it is difficult for others to detect and intervene with emotional mistreatment, as a covert form of mistreatment. Moreover, emotional mistreatment is very harmful and can produce long-term devastating damage to the mental health of older adults. Additionally, in order to intervene in the emotional mistreatment of older adults, the use of long-term family care and community care may play a role. In this paper, we discussed the mitigating effects of long-term community-based services on emotional mistreatment, which enable older adults to open up and communicate with others who can effectively detect and intervene in emotional mistreatment. We did not discuss the mitigating effect of long-term family care on emotional mistreatment in older adults, and we expect that long-term care by a broader range of family members could play a greater role in mitigating emotional mistreatment in older adults.

## 6. Influence and Limitations

In this study, older adults were not only affected by emotional mistreatment, but also by physical mistreatment. Both physical and emotional mistreatment adversely affected the mental health of older adults, which, in turn, reduced their life satisfaction. The expansion of family networks and social networks can help to reduce elder mistreatment and, thus, increase life satisfaction, but family networks and social networks can only work on the obvious level of physical mistreatment and cannot help with the difficult issue of emotional mistreatment. This suggests that the government, non-profit organizations, and other agencies should pay special attention to the emotional mistreatment of older adults, which is more concealed by traditional thinking.

Second, in terms of potential interventions, the use of direct intervention methods may be ineffective. Counseling services, for example, may be resisted by older adults when faced with direct external interventions, which, in turn, may reduce the exposure of their experiences of emotional mistreatment. One possible potential intervention mechanism is the provision of daily services, such as shopping, housework, or meal preparation services; these, on the one hand, can establish a close relationship with the older person over a longer time frame, and on the other hand, can provide in-depth communication with the older person to learn about their emotional mistreatment and the opportunity to intervene accordingly. Another potential intervention mechanism is the long-term care of broader family members, and research on this component will be carried out in depth in the future.

Although this study makes some contributions in the above areas, it still has some limitations. First, the dependent variable used in this paper is self-reported life satisfaction, which has a tendency to be overestimated compared to objective facts. Second, in this study, we found that only 6.47% of older adults had experienced at least one form of mistreatment, and the skewness and kurtosis of this paper’s indicators measuring physical and emotional mistreatment were high. This suggests that in future studies, a larger sample or better estimation methods might make the results more robust and credible. Third, in this study, one of the sub-questions of one of the items in the measure of “emotional mistreatment” contained physical violence due to the design of the questionnaire, which affected the distinction between physical and emotional mistreatment.

## 7. Conclusions

In this study, we focused on the influence of family networks and social networks on the life satisfaction of older adults, and explored mechanisms of reducing the physical and emotional mistreatment of elders. Through ordinary least squares, ordinal logistic regression, and mediated effects models, we came to the following conclusions.

First, family networks and social networks have significant positive effects on the life satisfaction of older adults, and the influence of the social network is greater. Second, both family networks and social networks enhance the life satisfaction of older adults by reducing physical mistreatment, but cannot effectively reduce the emotional mistreatment of older adults. The influence mechanism of family networks was more influential in enhancing the life satisfaction of older adults through the reduction in physical mistreatment. Third, in terms of measures to mitigate the emotional mistreatment of older adults, long-term community services may be effective, while direct psychological interventions may not have the desired effect due to resistance.

In terms of future research, the emotional mistreatment of older adults needs to receive more attention. In terms of interventions, broader community services need to be included in the study, and the effects of long-term care and attention at the family level should be tested in comparison.

## Figures and Tables

**Table 1:** Definition of variables and descriptive statistics.

Variable	Observations	Mean	Std	Min	Max
Family Networks	11,418	7.35	2.83	0	15
Social Networks	11,418	6.34	3.21	0	15
Life Satisfaction	11,306	3.78	0.87	1	5
Physical Mistreatment	11,418	0.03	0.22	0	3
Emotional Mistreatment	11,418	0.08	0.36	0	3
Age	11,418	71.45	7.37	60	108
Gender	11,418	0.50	0.50	0	1
Marriage	11,418	0.69	0.46	0	1
Religion	11,418	0.07	0.25	0	1
Ethnicity	11,418	0.95	0.22	0	1
Hukou	11,418	0.57	0.49	0	1
Education	11,418	2.18	0.98	1	5
Health	11,402	3.31	0.90	1	5
Years of Residence in the County	11,388	61.33	17.78	0	108

**Table 2:** Family Network and Social Network Scale questions.

	Question	None	1	2	3 to 4	5 to 8	9 or More
Family networks	How many family members/relatives do you see or contact at least once a month?	0	1	2	3	4	5
	How many family members/relatives are you comfortable talking to about your personal business?	0	1	2	3	4	5
	How many family members/relatives are available to help you when you need it?	0	1	2	3	4	5
Social networks	How many friends do you see or contact at least once a month?	0	1	2	3	4	5
	How many friends are you comfortable talking about your personal business with?	0	1	2	3	4	5
	How many friends can you count on to help you when you need it?	0	1	2	3	4	5

**Table 3:** Univariate regression analysis.

Variables	Life Satisfaction			
Family Networks	0.0088 ***	0.0060 **		
	(0.0029)	(0.0028)		
Social Networks			0.0204 ***	0.0122 ***
			(0.0026)	(0.0025)
Age		0.0005		0.0008
		(0.0013)		(0.0013)
Gender		−0.0284 *		−0.0283 *
		(0.0161)		(0.0160)
Marriage		0.0419 **		0.0403 **
		(0.0191)		(0.0191)
Religion		0.0897 ***		0.0906 ***
		(0.0317)		(0.0316)
Ethnicity		0.1050 ***		0.1090 ***
		(0.0337)		(0.0336)
Hukou		−0.0721 ***		−0.0689 ***
		(0.0170)		(0.0170)
Education		0.0299 ***		0.0282 ***
		(0.0089)		(0.0089)
Health		0.3020 ***		0.2990 ***
		(0.0102)		(0.0102)
Years of Residence in the County		0.0030 ***		0.0029 ***
		(0.0006)		(0.0005)
Constant	3.7120 ***	2.3660 ***	3.6470 ***	2.3250 ***
	(0.0226)	(0.1100)	(0.0187)	(0.1100)
Observations	11,273	11,233	11,273	11,233
R-squared	0.001	0.108	0.006	0.110

Note: Robust standard errors are in parentheses. The numbers above the parentheses are the unstandardized regression coefficients obtained from the ordinary least squares regression; *** *p* < 0.01, ** *p* < 0.05, * *p* < 0.1.

**Table 4:** Robustness test: marginal treatment effect of ordinal logistic regression.

Variables	Life Satisfaction				
	Value = 1	Value = 2	Value = 3	Value = 4	Value = 5
Family Networks	−0.0002 ***	−0.0010 ***	−0.0024 ***	0.0011 ***	0.0026 ***
	(0.0001)	(0.0004)	(0.0009)	(0.0004)	(0.0010)
Social Networks	−0.0003 ***	−0.0016 ***	−0.0039 ***	0.0017 ***	0.0041 ***
	(0.0001)	(0.0003)	(0.0008)	(0.0003)	(0.0008)
Age	−0.0000	−0.0001	−0.0002	0.0001	0.0003
	(0.0000)	(0.0002)	(0.0004)	(0.0002)	(0.0004)
Gender	0.0006	0.0032	0.0078	−0.0034	−0.0082
	(0.0004)	(0.0021)	(0.0051)	(0.0022)	(0.0054)
Marriage	−0.0008 *	−0.0043 *	−0.0106 *	0.0046 *	0.0111 *
	(0.0004)	(0.0024)	(0.0059)	(0.0025)	(0.0062)
Religion	−0.0021 ***	−0.0117 ***	−0.0287 ***	0.0124 ***	0.0302 ***
	(0.0008)	(0.0041)	(0.0100)	(0.0043)	(0.0105)
Ethnicity	−0.0024 ***	−0.0132 ***	−0.0324 ***	0.0140 ***	0.0340 ***
	(0.0008)	(0.0045)	(0.0111)	(0.0048)	(0.0116)
Hukou	0.0014 ***	0.0080 ***	0.0195 ***	−0.0084 ***	−0.0205 ***
	(0.0004)	(0.0022)	(0.0055)	(0.0024)	(0.0057)
Education	−0.0008 ***	−0.0046 ***	−0.0112 ***	0.0049 ***	0.0118 ***
	(0.0002)	(0.0012)	(0.0029)	(0.0013)	(0.0030)
Health	−0.0072 ***	−0.0405 ***	−0.0992 ***	0.0428 ***	0.1040 ***
	(0.0007)	(0.0018)	(0.0028)	(0.0021)	(0.0033)
Years of Residence in the County	−0.0001 ***	−0.0004 ***	−0.0009 ***	0.0004 ***	0.0009 ***
	(0.0000)	(0.0001)	(0.0002)	(0.0001)	(0.0002)
Observations	11,233	11,233	11,233	11,233	11,233

Note: Standard errors are in parentheses. The numbers above the parentheses are the unstandardized regression coefficients obtained from the ordinal logistic regression; *** *p* < 0.01, * *p* < 0.1.

**Table 5:** Test of the mediating effect of family networks.

Variables	Life Satisfaction	Physical Mistreatment	Life Satisfaction	Emotional Mistreatment	Life Satisfaction
Family Networks	0.0060 **	−0.0042 ***	0.0057 **	−0.0019	0.0058 **
	(0.0028)	(0.0008)	(0.0028)	(0.0013)	(0.0028)
Physical Mistreatment			−0.0815 **		
			(0.0328)		
Emotional Mistreatment					−0.1040 ***
					(0.0227)
Age	0.0005	0.0010 **	0.0006	0.0006	0.0006
	(0.0013)	(0.0004)	(0.0013)	(0.0006)	(0.0013)
Gender	−0.0284 *	0.0069	−0.0279 *	0.0126 *	−0.0272 *
	(0.0161)	(0.0044)	(0.0161)	(0.0071)	(0.0160)
Marriage	0.0419 **	0.0012	0.0420 **	−0.0119	0.0407 **
	(0.0191)	(0.0052)	(0.0191)	(0.0086)	(0.0190)
Religion	0.0897 ***	−0.0103	0.0888 ***	0.0167	0.0912 ***
	(0.0317)	(0.0072)	(0.0317)	(0.0141)	(0.0317)
Ethnicity	0.1050 ***	0.0189 ***	0.1070 ***	0.0589 ***	0.1110 ***
	(0.0337)	(0.0071)	(0.0337)	(0.0094)	(0.0338)
Hukou	−0.0721 ***	0.0000	−0.0720 ***	0.0098	−0.0711 ***
	(0.0170)	(0.0048)	(0.0170)	(0.0075)	(0.0169)
Education	0.0299 ***	−0.0081 ***	0.0292 ***	−0.0058	0.0293 ***
	(0.0089)	(0.0024)	(0.0089)	(0.0037)	(0.0089)
Health	0.3020 ***	0.0009	0.3020 ***	−0.0035	0.3020 ***
	(0.0102)	(0.0024)	(0.0102)	(0.0037)	(0.0102)
Years of Residence in the County	0.0030 ***	−0.0005 ***	0.0029 ***	−0.0009 ***	0.0029 ***
	(0.0005)	(0.0002)	(0.0005)	(0.0002)	(0.0005)
Constant	2.3660 ***	0.0209	2.3680 ***	0.0690	2.3740 ***
	(0.1100)	(0.0301)	(0.1110)	(0.0472)	(0.1110)
Observations	11,233	11,339	11,233	11,339	11,233
R-squared	0.108	0.006	0.108	0.004	0.110

Note: Robust standard errors are in parentheses. The numbers above the parentheses are the unstandardized regression coefficients obtained from the ordinary least squares; *** *p* < 0.01, ** *p* < 0.05, * *p* < 0.1.

**Table 6:** Test of the mediating effect of social networks.

Variables	Life Satisfaction	Physical Mistreatment	Life Satisfaction	Emotional Mistreatment	Life Satisfaction
Social Networks	0.0122 ***	−0.0028 ***	0.0120 ***	−0.0003	0.0122 ***
	(0.0025)	(0.0007)	(0.0025)	(0.0011)	(0.0025)
Physical Mistreatment			−0.0786 **		
			(0.0328)		
Emotional Mistreatment					−0.1050 ***
					(0.0227)
Age	0.0008	0.0009 **	0.0009	0.0006	0.0009
	(0.0013)	(0.0004)	(0.0013)	(0.0006)	(0.0013)
Gender	−0.0283 *	0.0072	−0.0277 *	0.0128 *	−0.0270 *
	(0.0160)	(0.0044)	(0.0160)	(0.0071)	(0.0160)
Marriage	0.0403 **	0.0008	0.0403 **	−0.0124	0.0389 **
	(0.0191)	(0.0052)	(0.0190)	(0.0086)	(0.0190)
Religion	0.0906 ***	−0.0108	0.0898 ***	0.0165	0.0922 ***
	(0.0316)	(0.0072)	(0.0317)	(0.0141)	(0.0316)
Ethnicity	0.1090 ***	0.0187 ***	0.1110 ***	0.0593 ***	0.1150 ***
	(0.0336)	(0.0071)	(0.0337)	(0.0094)	(0.0337)
Hukou	−0.0689 ***	−0.0002	−0.0688 ***	0.0100	−0.0679 ***
	(0.0170)	(0.0048)	(0.0170)	(0.0075)	(0.0170)
Education	0.0282 ***	−0.0082 ***	0.0276 ***	−0.0059	0.0276 ***
	(0.0089)	(0.0025)	(0.0089)	(0.0037)	(0.0089)
Health	0.2990 ***	0.0015	0.2990 ***	−0.0035	0.2990 ***
	(0.0102)	(0.0024)	(0.0102)	(0.0037)	(0.0102)
Years of Residence in the County	0.0029 ***	−0.0005 ***	0.0029 ***	−0.0009 ***	0.0028 ***
	(0.0005)	(0.0002)	(0.0005)	(0.0002)	(0.0005)
Constant	2.3250 ***	0.0117	2.3260 ***	0.0588	2.3320 ***
	(0.1100)	(0.0299)	(0.1100)	(0.0461)	(0.1100)
Observations	11,233	11,339	11,233	11,339	11,233
R-squared	0.110	0.005	0.110	0.004	0.112

Note: Robust standard errors are in parentheses. The numbers above the parentheses are the unstandardized regression coefficients obtained from the ordinary least squares; *** *p* < 0.01, ** *p* < 0.05, * *p* < 0.1.

**Table 7:** Mitigation role of community services.

Variables	Life Satisfaction			
Emotional Mistreatment	−0.3520 ***	−0.3480 ***	−0.3590 ***	−0.3510 ***
	(0.0395)	(0.0388)	(0.0403)	(0.0408)
Emotional Mistreatment * The community provides escort services for medical appointments	0.2450 ***			
	(0.0894)			
Emotional Mistreatment * The community provides home-based chore services		0.3570 ***		
		(0.0811)		
Emotional Mistreatment * Community meal table or meal delivery service for seniors			0.3470 ***	
			(0.0939)	
Emotional Mistreatment * Community-based counseling services				0.1760 *
				(0.0998)
Age	0.0001	0.0001	0.0003	0.0001
	(0.0019)	(0.0019)	(0.0019)	(0.0019)
Gender	0.0062	−0.0005	−0.0063	0.0022
	(0.0238)	(0.0235)	(0.0229)	(0.0235)
Marriage	0.0363	0.0345	0.0461 *	0.0378
	(0.0277)	(0.0277)	(0.0271)	(0.0276)
Religion	0.0753	0.0870 *	0.0725	0.0667
	(0.0491)	(0.0474)	(0.0484)	(0.0485)
Ethnicity	0.1010 **	0.1160 ***	0.1310 ***	0.0970 **
	(0.0435)	(0.0435)	(0.0436)	(0.0434)
Hukou	0.0519 **	0.0222	0.0007	0.0334
	(0.0255)	(0.0258)	(0.0259)	(0.0261)
Education	0.0026	0.0140	0.0130	0.0054
	(0.0133)	(0.0132)	(0.0129)	(0.0133)
Health	0.2960 ***	0.2980 ***	0.2900 ***	0.2820 ***
	(0.0152)	(0.0153)	(0.0151)	(0.0154)
Years of Residence in the County	0.0027 ***	0.0026 ***	0.0020 ***	0.0027 ***
	(0.0008)	(0.0008)	(0.0008)	(0.0008)
Constant	2.5130 ***	2.5190 ***	2.5920 ***	2.6030 ***
	(0.1650)	(0.1670)	(0.1620)	(0.1660)
Observations	5190	5319	5392	5137
R-squared	0.118	0.119	0.124	0.112

Note: Robust standard errors are in parentheses. The numbers above the parentheses are the unstandardized regression coefficients obtained from the ordinary least squares; *** *p* < 0.01, ** *p* < 0.05, * *p* < 0.1.

## Data Availability

Not applicable.

## Appendix A

**Table A1:** Correlation analysis.

	Life Satisfaction	Family Networks	Social Networks	Physical Mistreatment	Emotional Mistreatment	Age	Gender	Marriage	Religion	Ethnicity	Hukou	Education	Health	Years of Residence in the County
Life Satisfaction	1													
Family Networks	0.017 *	1												
Social Networks	0.068 ***	0.645 ***	1											
Physical Mistreatment	−0.040 ***	−0.048 ***	−0.035 ***	1										
Emotional Mistreatment	−0.051 ***	−0.016 *	−0.009	0.739 ***	1									
Age	−0.031 ***	−0.006	−0.071 ***	0.020 **	0.002	1								
Gender	0.011	−0.001	0.012	0.010	0.007	−0.020 **	1							
Marriage	0.056 ***	0.051 ***	0.070 ***	−0.007	−0.012	−0.322 ***	0.211 ***	1						
Religion	0.028 ***	0.005	−0.003	0.002	0.011	0.017 *	−0.020 **	0.018 *	1					
Ethnicity	0.025 ***	−0.017 *	−0.024 **	0.030 ***	0.034 ***	−0.015	−0.006	0.035 ***	−0.071 ***	1				
Hukou	−0.067 ***	−0.054 ***	−0.076 ***	0.014	0.013	−0.020 **	0.038 ***	−0.044 ***	−0.030 ***	−0.102 ***	1			
Education	0.079 ***	0.068 ***	0.104 ***	−0.033 ***	−0.015	−0.213 ***	0.139 ***	0.186 ***	−0.022 **	0.048 ***	−0.371 ***	1		
Health	0.317 ***	0.015	0.090 ***	−0.010	−0.012	−0.157 ***	0.050 ***	0.116 ***	0.016 *	−0.019 **	−0.048 ***	0.129 ***	1	
Years of Residence in the County	0.028 ***	0.002	−0.013	−0.024 ***	−0.032 ***	0.422 ***	0.067 **	−0.126 ***	−0.087 ***	−0.016 *	0.140 ***	−0.211 ***	−0.051 ***	1

Note: Numbers represent correlation coefficients, * represents 90% confidence level, ** represents 95% confidence level, and *** represents 99% confidence level.
